# Severe COVID-19 in Hospitalized Carriers of Single *CFTR* Pathogenic Variants

**DOI:** 10.3390/jpm11060558

**Published:** 2021-06-15

**Authors:** Margherita Baldassarri, Francesca Fava, Chiara Fallerini, Sergio Daga, Elisa Benetti, Kristina Zguro, Sara Amitrano, Floriana Valentino, Gabriella Doddato, Annarita Giliberti, Laura Di Sarno, Maria Palmieri, Miriam Lucia Carriero, Diana Alaverdian, Giada Beligni, Nicola Iuso, Francesco Castelli, Eugenia Quiros-Roldan, Mario Umberto Mondelli, Rosalba Miceli, Elisa Frullanti, Simone Furini, Francesca Mari, Alessandra Renieri, Chiara Gabbi, on behalf of the GEN-COVID Multicenter Study

**Affiliations:** 1 Medical Genetics, University of Siena, 53100 Siena, Italy; margherita.baldassarri@dbm.unisi.it (M.B.); francesca.fava@dbm.unisi.it (F.F.); fallerini2@unisi.it (C.F.); sergio.daga@dbm.unisi.it (S.D.); floriana.valentino@dbm.unisi.it (F.V.); gabriella.doddato@dbm.unisi.it (G.D.); giliberti@student.unisi.it (A.G.); laura.disarno@dbm.unisi.it (L.D.S.); maria.palmieri@dbm.unisi.it (M.P.); miriam.carriero@dbm.unisi.it (M.L.C.); diana.alaverdian@dbm.unisi.it (D.A.); giada.beligni@dbm.unisi.it (G.B.); nicolaiuso94@gmail.com (N.I.); elisa.frullanti@dbm.unisi.it (E.F.); francesca.mari@dbm.unisi.it (F.M.); 2 Med Biotech Hub and Competence Center, Department of Medical Biotechnologies, University of Siena, 53100 Siena, Italy; elisa.benetti@dbm.unisi.it (E.B.); kristina.zguro@student.unisi.it (K.Z.); simone.furini@unisi.it (S.F.); 3 Genetica Medica, Azienda Ospedaliero-Universitaria Senese, 53100 Siena, Italy; sara.amitrano@dbm.unisi.it; 4 Department of Infectious and Tropical Diseases, University of Brescia and ASST Spedali Civili Hospital, 25123 Brescia, Italy; francesco.castelli@unibs.it (F.C.); maria.quirosroldan@unibs.it (E.Q.-R.); 5 Department of Internal Medicine and Therapeutics, University of Pavia, 27100 Pavia, Italy; marioumberto.mondelli@unipv.it; 6 Division of Infectious Diseases and Immunology, Fondazione IRCCS Policlinico San Matteo, 27100 Pavia, Italy; 7 Clinical Epidemiology and Trial Organization, Department of Applied Research and Technological Development, Fondazione IRCCS Istituto Nazionale Tumori di Milano, 20133 Milan, Italy; rosalba.miceli@istitutotumori.mi.it; 8 Independent Researcher, 20145 Milan, Italy

**Keywords:** CF carrier screening, host genetics, COVID-19, CFTR

## Abstract

The clinical presentation of COVID-19 is extremely heterogeneous, ranging from asymptomatic to severely ill patients. Thus, host genetic factors may be involved in determining disease presentation and progression. Given that carriers of single cystic fibrosis (CF)-causing variants of the *CFTR* gene—CF-carriers—are more susceptible to respiratory tract infections, our aim was to determine their likelihood of undergoing severe COVID-19. We implemented a cohort study of 874 individuals diagnosed with COVID-19, during the first pandemic wave in Italy. Whole exome sequencing was performed and validated CF-causing variants were identified. Forty subjects (16 females and 24 males) were found to be CF-carriers. Among mechanically ventilated patients, CF-carriers were more represented (8.7%) and they were significantly (*p* < 0.05) younger (mean age 51 years) compared to noncarriers (mean age 61.42 years). Furthermore, in the whole cohort, the age of male CF-carriers was lower, compared to noncarriers (*p* < 0.05). CF-carriers had a relative risk of presenting an abnormal inflammatory response (CRP ≥ 20 mg/dL) of 1.69 (*p* < 0.05) and their hazard ratio of death at day 14 was 3.10 (*p* < 0.05) in a multivariate regression model, adjusted for age, sex and comorbidities. In conclusion, CF-carriers are more susceptible to the severe form of COVID-19, showing also higher risk of 14-day death.

## 1. Introduction

The severe acute respiratory syndrome coronavirus 2 (SARS-CoV-2) is one of the members of the Coronaviridae family [1] that, since December 2019, has caused pandemic outbreaks of human infections [2].

The clinical presentation of the coronavirus disease (COVID-19) is particularly heterogeneous ranging from asymptomatic [3] to critically ill patients [4]. Beside the majority of subjects presenting with mild symptoms, the reported severe cases are characterized by bilateral pneumonia associated with an extreme inflammatory response [5], hepatitis [6], pancreatic involvement [7], cardiac injury, renal failure, neurologic and thromboembolic complications [1,5,8,9].

While age and the presence of pre-existing conditions, like diabetes and cardiovascular diseases, explain to some extent the worse prognosis of certain patients [4,10], it is conceivable that host genetic factors may contribute to disease presentation and progression. In this direction, it has been identified a genetic locus, corresponding to the ABO-blood group, that is highly represented in COVID-19 patients with respiratory failure [11]. Furthermore, deletion in the gene encoding for the NKG2C receptor (*KLRC2*) that mediates the activation of natural killer cells, has been shown to be associated with the severe form of COVID-19 [12] and monogenic defects of immunity to SARS-CoV-2, appeared in 3.5% of patients with life-threatening COVID-19 pneumonia [13]. Additional studies aimed at detangling the host genetic complexity in determining COVID-19 susceptibility and clinical presentation are ongoing, including a global initiative [14] to which our GEN-COVID consortium belongs [15,16,17,18,19,20].

GEN-COVID is a multicenter observational study, conducted in 28 hospitals, primary care centers and public health units in Italy, performing extensive genetic and clinical characterizations of patients affected by COVID-19 [15,16,17,18,19,20]. As part of this national effort, we conducted the present study, under the hypothesis that a subset of individuals carrying single pathogenic variants of the cystic fibrosis transmembrane conductance regulator (*CFTR*) gene is more susceptible to the most critical form of COVID-19. CFTR is a chloride and bicarbonate channel expressed on the apical membrane of epithelial cells, mainly in lung, liver, pancreas and intestine [21], where also ACE2 (angiotensin-converting enzyme 2), the entry receptor for SARS-CoV-2 [22], is localized [23]. When both copies of the *CFTR* gene are mutated, patients are affected by cystic fibrosis (CF), a genetic disease characterized by high viscosity of secreted fluids and by an abnormal inflammatory response, independent but aggravated by infections [24], leading to respiratory failure and premature death [21]. Carriers of one CF-causing variant, have a reduction in CFTR expression and function, depending on the type of the pathogenic variant, and have high risk of developing CF-related conditions [25]. In particular, they are known to be significantly more susceptible to airway and sinus infections, pneumonia, pancreatic injury and hepatitis [25,26]: all conditions described in the severe form of COVID-19 [4,6,7].

Therefore, the objective here was to identify, among patients enrolled in the GEN-COVID cohort [15,16,17,18,19,20], those that are carriers of single pathogenic variants of the *CFTR* gene and evaluate their clinical course, in order to determine to what extent CFTR impairment contributes to COVID-19 susceptibility and severity.

## 2. Materials and Methods

Patient population. Patients affected by COVID-19 were recruited through the GEN-COVID multicenter study (NCT04549831) in 28 hospitals, local healthcare units and departments of preventive medicine in Italy from 8 April to 30 June 2020 [15,16,17,18,19,20]. All the enrolled patients were adults (aged ≥18 years) with SARS-CoV-2 infection confirmed by reverse transcriptase-polymerase chain reaction (PCR) assay on nasopharyngeal swab. The clinical severity of COVID-19 was assessed using a modified version of the WHO COVID-19 Outcome Scale [27], identifying the following six categories: 1, death; 2, hospitalized receiving invasive mechanical ventilation; 3, hospitalized, receiving continuous positive airway pressure (CPAP) or bilevel positive airway pressure (BiPAP) ventilation; 4, hospitalized, receiving low-flow supplemental oxygen; 5, hospitalized, not receiving supplemental oxygen; 6, not hospitalized. The GEN-COVID study was approved by the Institutional Review Board (IRB) of the Siena University Hospital (Protocol n. 16917, 16 March 2020) and by the local IRBs of all the recruiting hospitals involved. Patients or legally authorized representatives provided informed consent for participating in the study.

Whole Exome sequencing analysis and identification of *CFTR* pathogenic variants. Whole Exome sequencing with at least 97% coverage at 20× was performed using the Illumina NovaSeq6000 System (Illumina, San Diego, CA, USA). Library preparation was performed using the Illumina Exome Panel (Illumina) following manufacturer’s protocol. Library enrichment was tested by qPCR, and the size distribution and concentration were determined using Agilent Bioanalyzer 2100 (Agilent Technologies, Santa Clara, CA, USA). The Novaseq6000 System (Illumina) was used for DNA sequencing through 150 bp paired-end reads. Variants calling was performed according to the GATK4 best practice guidelines for joint calling, using BWA for mapping. The results of the joint call were annotated by ANNOVAR, and variants in the *CFTR* gene already reported as disease causing in the “CFTR2 Database-Clinical and Functional Translation of CFTR” (https://cftr2.org/, last accessed on 12 June 2021) and/or in ClinVar database (https://www.ncbi.nlm.nih.gov/clinvar/, last accessed on 12 June 2021) were selected.

Statistical methods. Wilcoxon rank-sum test was applied to compare differences between two groups for not-normally distributed values while an independent Student’s *t*-test was used for normally distributed ones. The binary association between the presence of CF-causing variant and the COVID-19 Outcome scale was assessed using the Cochran–Armitage test for trend. The Fisher–Freeman–Halton test or Fisher exact test were used when testing association between categorical variables [28] as indicated. Relative risk (RR) of severe clinical outcome was estimated in carriers vs noncarriers, with and without adjustment for patient age. To define severe outcome, the following parameters were set: PaO_2_/FiO_2_ ≤ 250 indicating severe respiratory impairment; C Reactive Protein (CRP) ≥ 20 mg/dL; ALT and AST ≥ 40 U/L; LDH ≥ 400 U/L. Overall survival (OS) curves were estimated with the Kaplan-Meier method and compared with the log-rank test. OS time was calculated from the date of patient admission to the day of death from any cause related to COVID-19 or the discharge day for alive patients. The nonhospitalized patients were included in the analyses with an OS time equal to 1 day, as they were enrolled the same day of the nasopharyngeal swab execution and limited follow-up was performed. The same strategy was adopted for hospitalized patients lost to follow-up after the hospitalization. Cox regression analysis was used for univariable and multivariable analyses of association between OS and carrier status. Due to the low number of OS events, in multivariable Cox analysis the adjustment for confounding variables (i.e., patients’ age and sex, chronic conditions, such as hypertension, diabetes, asthma/ chronic obstructive pulmonary disease (COPD), congestive heart failure (CHF) and coronary artery disease (CAD), malignancy, hypothyroidism, obesity, and admission period less than or greater or equal to 15 April 2020) was operated by means of a score beforehand estimated as the linear predictor from a Cox model including all the confounding variables. The admission period less than or greater or equal to 15 April 2020 was chosen as a proxy for the different hospital admission conditions in the very first pandemic period, that severely hit Italy, vs the most recent one. Continuous variables were presented as mean ± SD or median (interquartile range, IQR) as indicated.

Statistical analyses were performed with SAS (SAS Institute, Cary, NC, USA), R- (R-Foundation for Statistical Computing, Vienna, Austria) and STATA/IC 15.0 software on a mac workstation. Statistical significance was set at 5% level.

## 3. Results

### 3.1. Cohort Patients

Eight-hundred and seventy-four patients (43.13% females, 56.75% males) were studied (Table 1). The mean age was 59.9 years (SD, 15.64) and it was significantly higher in all the hospitalized categories compared to the nonhospitalized one (Table 1). In all the hospitalized categories there was a significantly higher prevalence of males compared to category 6, i.e., patients not requiring hospitalization; in particular, 74.29% of the subjects that underwent invasive mechanical ventilation were males (*p* < 0.001) (Table 1). The most frequent associated chronic conditions are listed in Table 1 and Supplementary Table S1.

### 3.2. Identified CFTR Pathogenic Variants

Seventeen CF-causing variants were identified in 41 COVID-19 patients (Supplementary Table S2). The majority of carriers (n = 13) had a genomic deletion of three base pairs resulting in the loss of phenylalanine at amino acid position 508 of the CFTR protein (Supplementary Table S2). Nobody was carrier of the TG12-5T polymorphism, nor the TG13-5T known to reduce CFTR function [29].

### 3.3. Demographics and Clinical Characteristics of CF Carriers

Forty patients (4.58% of the whole cohort), 26 males (65%) and 14 females (35%), were identified as carriers of one CF-causing variant in the *CFTR* gene while one patient (male, 52 years) was found to have two pathogenic variants (Supplementary Table S2). The following analyses were performed including only carriers of single variants. Carriers’ prevalence increased with the worsening of COVID-19 Outcome scale, peaking to 8.7% in patients undergoing invasive mechanical ventilation and being 2.54% in patients receiving low oxygen flow (Table 2). This trend was significantly evident (*p* < 0.0001) for patients younger than 50 years; among them, 25% of those receiving invasive mechanical ventilation were carriers. Patients carrying CF-causing variants were prevalently males in all the outcome categories except for category 1 and 3; in particular, 83.33% of carriers receiving invasive mechanical ventilation were males (Supplementary Table S3).

Overall, male carriers were significantly younger (mean age ± SD: 53.08 ± 18.8 years) compared to noncarriers (mean age ± SEM: 60.37 ± 14.57 years) (*p* < 0.05) and they underwent invasive mechanical ventilation at a mean age of 51 years while noncarriers at 61.42 years (*p* < 0.05) (Table 3). The prevalence of comorbidities was not different between the two groups except for hypertension that was higher in not carriers (Supplementary Table S5).

Patients were monitored during the whole course of the hospitalization and the worse clinical and biochemical parameters were registered. The RR of undergoing respiratory impairment (PaO_2_/FiO_2_ ≤ 250) for carriers was 1.54 (95% CI: 0.99–1.76; *p* = 0.053) suggesting that they were prone to develop an acute respiratory distress syndrome (ARDS) associated with widespread inflammation. Indeed, their RR of having levels of CRP ≥ 20 mg/dL (twice the upper limit) was 1.69 (95% CI: 1.06–2.29; *p* = 0.03) (Table 4). No significant higher risk of increased liver enzymes, and LDH was registered for carriers. All the carriers for whom the serum pancreatic profile was available (n = 3) showed a marked hyperlipasemia (serum lipase ≥ 360 U/L).

### 3.4. Mortality in Carriers of CF-Causing Variants

Among the 874 patients included in the study, 55 patients (6.29%) underwent exitus. Their mean age was 75.73 years (SD, 10.98) and 54.55% of them were males (Table 1). Fatal cases (category 1) showed a higher prevalence of hypertension (25.45%), cardiovascular diseases (14.55%), asthma and COPD (9.09%), malignancy (7.27%) compared to the nonhospitalized category (Supplementary Table S1).

**Figure 1:**
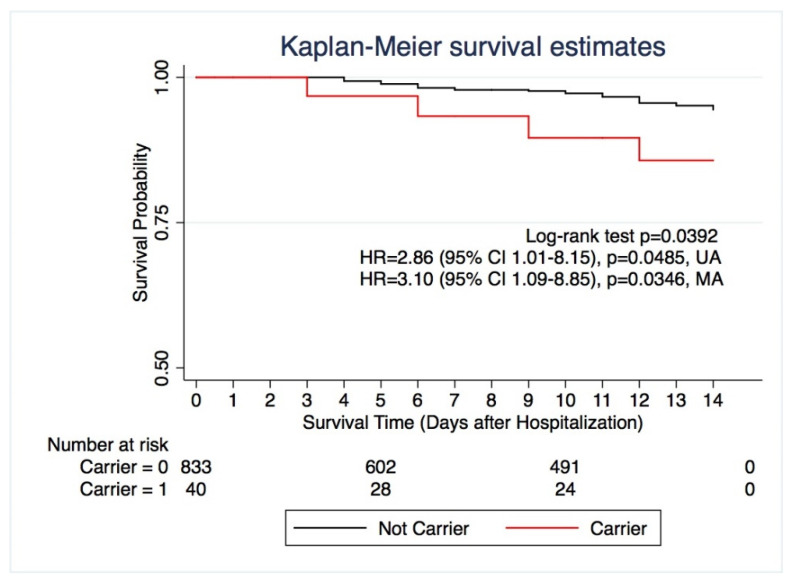
Survival Study: Kaplan–Meier 14-day survival study comparing carriers of single CF-causing variants (red) and noncarriers (black). Log-rank test *p* = 0.0392; Univariable Cox analysis (UA) HR = 2.86; 95% CI 1.01–8.15; *p* = 0.0485. Multivariable Cox analysis (MA) with adjustment for confounding variables HR = 3.10; 95% CI, 1.09–8.85; *p* = 0.0346.

In an univariable Cox analysis of independent risk factors related to fatal outcome at day 14 after hospital admission, CF-carrier status showed HR of 2.86 (95% CI: 1.01–8.15; *p* = 0.04); after adjustment for confounding variables (age, sex, comorbidities) in a multivariate Cox analysis the HR for CF carriers raised to 3.10 (95% CI: 1.09–8.85; *p* = 0.03) (Figure 1; Supplementary Table S5). The Kaplan–Meier estimates of 14-day survival were 85.42% (95% CI, 65.51 to 94.30) for the carriers and 93.63 % (95% CI, 90.70 to 95.66) for the noncarriers (*p* = 0.03 Log rank test) (Figure 1).

When considering only hospitalized patients, the 14-day survival was 85.15% (95% CI, 72.66–99.79) for carriers and 94.04 % (95% CI, 91.93 to 96.19) for the noncarriers (*p* = 0.0447 Log rank test). The HR of 14-day death for carriers was 3.06 (95% CI, 1.07–8.73; *p* = 0.0365) after adjustment for confounding variables.

Other independent factors for early mortality (14 days after admission) were: age ≥ 75 years (HR: 4.53; 95% CI, 2.27–9.04; *p* < 0.0001); LDH ≥ 400 (HR: 3.64; 95% CI, 1.30–10.22; *p* = 0.0141) (Supplementary Table S5). Univariable Cox analysis of independent risk factors related to fatal outcome at day 28 and 60 after hospitalization, showed a HR for CF carriers equal to 1.97 (95% CI: 0.70–5.5; *p* = 0.1978) and 1.70 (95% CI: 0.61–4.73; *p* = 0.3087) respectively (Supplementary Table S5).

## 4. Discussion

In the present study we described the peculiarity of the CF-carriers in-hospital clinical course, characterized by high inflammatory response, severe respiratory impairment and higher risk of 14-day in-hospital mortality.

Carriers of CF-causing variants are known to have a reduction in CFTR function of about 50% the physiological level [30,31]. Although these individuals do not have CF, they are more susceptible to numerous CF-related diseases like pancreatitis, hepatitis and respiratory tract infections [25,26,32]. In our cohort, hospitalized CF carriers develop indeed a form of COVID-19 more likely characterized by acute respiratory distress syndrome (PaO_2_/FiO_2_ ratio ≤ 250 mmHg), high inflammatory response (CRP ≥ 20 mg/dL), and, for some of them hyperlipasemia. Moreover, carriers undergoing invasive mechanical ventilation have a mean age of 51 years, being significantly younger than noncarriers in the same clinical category. Thus, those evidences suggest that CF carriers may be prone also to develop a severe manifestation of COVID-19, and even at a younger age compared to noncarriers.

In our cohort the majority (83.33%) of CF carriers mechanically ventilated were males and in all the hospitalized categories there was a higher prevalence of male individuals. Such a result confirms a world trend that identifies the male sex as a relevant risk factor for severe COVID-19 [33,34]. While our GEN-COVID consortium has shown that male individuals carrying longer androgen receptor polyQ alleles are more critically ill [19], the high expression of ACE2, the entry receptor of SARS-CoV-2 [22], in testes could also be considered a putative factor determining gender susceptibility to the severe form of the disease [35].

Although numerous studies are now ongoing to clearly understand the complex pathogenesis of severe COVID-19, the respiratory impairment seems to be triggered by both a direct cytotoxic action of SARS-CoV-2 on airway cells and by an abnormal self-perpetuating inflammatory response [5,36]. ACE2 is known to exert anti-inflammatory effects by counterbalancing the proinflammatory action of the angiotensin-converting enzyme (ACE). After the binding virus-receptor, ACE2 expression in the lung is reduced, because of its internalization, leading to a proinflammatory cascade of cytokines through the NF-kB signaling and to a consequent lung injury [36,37]. Such pathogenic mechanism of action may occur also in other organs, expressing ACE2 receptors, that are affected by COVID-19, like the gastro-intestinal tract and the pancreas [4]. Indeed, 12 to 17% of patients with COVID-19 develop an elevation of pancreatic enzymes [7,38], as some CF carriers in our cohort showing elevated levels of lipase.

The predisposition of CF-carriers to severe COVID-19, also hypothesized by others [39], is probably related to multiple factors: i. the acidification of the airway surface liquid that impairs immune response [40]; ii. a reduced *CFTR* function [30,31] that upregulates the proinflammatory signaling, and that is associated with a deficiency in pro-solving mediators, known to promote the resolution of the inflammation [24] and iii. an accumulation of misfolded *CFTR* that may trigger NF-kB signaling [39]. Interestingly CFTR and ACE2 are known to colocalize in numerous epithelial cells, especially in the respiratory tract [23]. Thus, it is conceivable that in CF carriers the concomitant downregulation of CFTR and ACE2—having both events a proinflammatory effect—may lead to a more severe COVID-19 clinical presentation. While the hypothesized mechanisms are present, even to a larger extent, in carriers of two CF-causing variants, it’s interesting to notice that CF patients undergo, instead, a mild form of COVID-19 [41,42,43]. The explanation may lay the fact that CF patients, while costumed to always wear protective masks, are often treated with modulators that re-establish CFTR function or with other drugs, like azithromycin, that may protect against infections [41,42]. Detangling the role of CFTR in COVID-19 pathogenesis would for sure help to better understand such deadly disease, considering also that a previous GWAS study has described an intronic variant in the *CFTR* gene to be highly represented in patients with pneumonia [44].

One year after the first report on a novel coronavirus disease, the registered deaths have been more than 1.8 million worldwide [2]. Here we propose a novel survival study encompassing, not only demographics and clinical parameters of the patients but also their genetic profile related to *CFTR*. In our model, while we confirmed [45] age ≥ 75 and AST ≥ 40 U/L to be relevant risk factors for mortality at all the studied time points and at 28/60-days respectively, we identified the status of CF carrier, LDH ≥ 400, age ≥ 75—to be determinants of mortality at day 14. In particular, being a carrier of known CF-causing variants appears to be a relevant factor (HR, 3.10, CI, 1.09–8.85) determining early mortality after adjustments for age, sex and comorbidities. Importantly, at the later time-points (day-28 and day-60) CF carrier status does not appear to be anymore a risk factor for death. Such a finding reveals that COVID-19 mortality is determined by time-dependent factors and that CFTR-related early events like cytokine storm may be responsible for early death.

The current study is limited to the Caucasian race, predominant in the Italian population. However, in our opinion those findings may be transferable to the other ethnicities, considering that in-hospital mortality does not appear to differ between white and black patients [46]. A further limitation of our study sits in the number of carriers studied (n = 40) that although resulting from a cohort of 874 patients, doesn’t allow more complex epidemiological analysis. Finally, as indicated in the methods section, the follow-up of not hospitalized patients is restricted to the initial stage of the disease.

## 5. Conclusions

In conclusion, while more studies should be performed to understand the role of *CFTR* in COVID-19 pathogenesis, in our opinion, the presented results may have relevant and immediate clinical implications. Indeed, CF is one of the most frequent genetic diseases in Caucasians with an estimated prevalence of one per 25/30 individuals [21] and the numerous people are CF carriers, considering also the effective CF-screening programs ongoing globally [47]. Thus, the status of CF carrier, given its high prevalence, should be investigated in COVID-19 hospitalized patients in order to identify subjects that, being at risk of severe disease, would benefit of intensive surveillance and personalized therapy.

## Figures and Tables

**Table 1:** Demographic and clinical characteristics of the enrolled patients.

	Whole Cohort (n = 874)	Female (n = 377, 43.13%)	Male (n = 496, 56.75%)
**Age, mean ± SD (year)**	59.9 ± 15.64	59.78 ± 14.89	59.99 ± 16.6
Cat. 1 (Death)	75.73 ± 10.98 §	77.4 ± 12.50	74.33 ± 9.53
Cat. 2 (Invasive mechanical ventilation)	60.53 ± 12.19 §	60 ± 13.86	60.71 ± 11.69
Cat. 3 (CPAP/BiPAP)	63.62 ± 12.46 §	66.91 ± 13.53	62.24 ± 11.79 °
Cat. 4 (Hospitalized with low flow oxygen)	65.50 ± 14.4 §	68.08 ± 14.75	63.77 ± 13.93 °
Cat. 5 (Hospitalized without oxygen)	55.74 ± 15.89 §	54.9 ± 16.85	56.5 ± 15.06
Cat. 6 (Not hospitalized)	47.68 ± 12.01	48.38 ± 10.74	46.64 ± 13.66
**COVID-19 Outcome Scale, No. (%)**			
Cat. 1 (Death)	55 (6.29)	25 (45.45)	30 (54.55) *
Cat. 2 (Invasive mechanical ventilation)	70 (8.01)	18 (25.71)	52 (74.29) **
Cat. 3 (CPAP/BiPAP)	146 (16.7)	43 (29.45)	103 (70.55) **
Cat. 4 (Hospitalized with low flow oxygen)	276 (31.58)	111 (40.22)	165 (59.64) **
Cat. 5 (Hospitalized without oxygen)	122 (13.96)	58 (47-54)	64 (52.46) *
Cat. 6 (Not hospitalized)	205 (23.45)	122 (59.51)	83 (40.49)
**Chronic Conditions, No. (%)**			
Hypertension	243 (27.8)	93 (38.27)	150 (61.63)
Diabetes	105 (12.22)	44 (41.9)	61 (58.1)
Asthma and COPD	72 (8.64)	33 (45.83)	39 (54.17)
CHF and CAD	68 (8)	21 (31.34)	47 (69.11) °
Malignancy	65 (7.24)	35 (53.85)	30 (46.15)
Hypothyroidism	42 (4.8)	29 (69.05) °°	13 (30.95)
Obesity	29 (3.35)	13 (44.83)	16 (55.17)

§ *p* < 0.0001 vs. Cat. 6, *t* Test; * *p* < 0.05; ** *p* < 0.001 vs Cat. 6, Fisher exact test; **°** *p* = 0.05; **°°** *p* < 0.01 vs. other sex, *t* Test or Fisher exact; CPAP: continuous positive airway pressure; BiPAP: bilevel positive airway pressure; CHF: congestive heart failure; CAD: coronary artery disease; COPD: chronic obstructive pulmonary disease. Percentages in column 2 indicate the prevalence in the whole cohort while in column 3 and 4 they refer to the prevalence of males and females in the group indicated in the corresponding line (i.e., COVID category or chronic condition).

**Table 2:** Carriers of single *CFTR* pathogenic variants.

	Total Cohort	Age < 50 years (n = 225)	Age ≥ 50 years (n = 648)
**Total Carriers, No.**	40	16	24
**Carriers by COVID-19 Outcome Scale, No. (%)**			
Cat. 1 (Death; n = 55)	4 (7.1)	0	4 (7.1)
Cat. 2 (Invasive mechanical ventilation; n = 70)	6 (8.7) *	3 (25) **	3 (5.3)
Cat. 3 (CPAP/BiPAP; n = 146)	8 (5.5)	2 (10)	6 (4.8)
Cat. 4 (Hospitalized, with low flow oxygen; n = 275)	7 (2.5)	2 (5.9)	5 (2.1)
Cat. 5 (Hospitalized, without oxygen; n = 122)	5 (4.1)	2 (4.7)	3 (3.8)
Cat. 6 (Not hospitalized; n = 205)	10 (4.9)	7 (6)	3 (3.4)

* *p* < 0.05 vs category 4 by Fisher’s exact test; ** *p* < 0.0001 by Chi-square test for trend.

**Table 3:** Demographics of carriers vs noncarriers.

	Not Carriers (n = 833)	Carriers (n = 40)
**Age, mean ± SD**		
All	60.09 ± 15.51	55.85 ± 17.88
Female	59.73 ± 16.66	61 ± 15.32
Male	60.37 ± 14.57	53.08 ± 18.8 §
Cat. 1 (Death)	75.67 ± 11.13	76.5 ± 10.38
Cat. 2 (Invasive mechanical ventilation)	61.42 ± 10.99	51.00 ± 20.21 *
Cat. 3 (CPAP/BiPAP)	63.64 ± 12.08	63.25 ± 19
Cat. 4 (Hospitalized with low flow oxygen)	65.77 ± 14.28	57.28 ± 17.74
Cat. 5 (Hospitalized without supplemental oxygen)	55.75 ± 16.13 °	55.40 ± 9.55 #
Cat. 6 (Not hospitalized)	47.88 ± 11.95 °°	43.80 ± 13.19 ##

* p < 0.05 vs Cat.2 noncarriers; # *p* < 0.05; ## *p* < 0.001 vs Cat.1 carriers; ° *p* < 0.05; °° *p* < 0.001 vs. Cat.1 noncarriers; **§** *p* < 0.05 vs noncarriers.

**Table 4:** Outcome RR for carriers of CF-causing variants adjusted for age.

Outcome	Level	RR	95% CI
PaO_2_/FIO_2_ ratio	≤250	1.54 *	0.99–1.76
Invasive mechanical ventilation/CPAP/BiPAP	yes vs no	1.50	0.97–2.07
C-reactive protein level (mg/dL)	≥20	1.69 **	1.06–2.29

* *p* = 0.05; ** *p* = 0.03.

## Data Availability

Data are available upon reasonable request to A.R.

## Supplementary Materials

The following are available online at https://www.mdpi.com/article/10.3390/jpm11060558/s1, Table S1: Prevalence of comorbidities by COVID-19 outcome scale and sex; Table S2: CF-causing variants in COVID-19 patients; Table S3: Carriers of CFTR Pathogenic Variants; Table S4: Prevalence of comorbidities by carrier status; Table S5: Univariable Cox Analysis of risk factors related to fatal outcome.

## Appendix A

GEN-COVID Multicenter Study Members (https://sites.google.com/dbm.unisi.it/gen-covid): Rossella Tita^3^, Mirella Bruttini^1,2,3^, Susanna Croci^1,2^, Ilaria Meloni^1,2^, Anna Maria Pinto^3^, Maria Antonietta Mencarelli^3^, Caterina Lo Rizzo^3^, Valentina Perticaroli^1,2,3^, Francesca Montagnani^2,9^, Mario Tumbarello^2^^,9^, Gabriele Inchingolo^1,2^, Massimiliano Fabbiani^9^, Barbara Rossetti^9^, Giacomo Zanelli^2,9^, Elena Bargagli^10^, Laura Bergantini^10^, Miriana D’Alessandro^10^, Paolo Cameli^10^, David Bennett^10^, Federico Anedda^11^, Simona Marcantonio^11^, Sabino Scolletta^11^, Federico Franchi^11^, Maria Antonietta Mazzei^12^, Susanna Guerrini^12^, Edoardo Conticini^13^, Luca Cantarini^13^, Bruno Frediani^13^, Danilo Tacconi^14^, Chiara Spertilli Raffaelli^14^, Marco Feri^15^, Alice Donati^15^, Raffaele Scala^16^, Luca Guidelli^16^, Genni Spargi^17^, Marta Corridi^17^, Cesira Nencioni^18^, Leonardo Croci^18^, Gian Piero Caldarelli^19^, Maurizio Spagnesi^20^, Paolo Piacentini^20^, Maria Bandini^20^, Elena Desanctis^20^, Silvia Cappelli^20^, Anna Canaccini^21^, Agnese Verzuri^21^, Valentina Anemoli^21^, Agostino Ognibene^22^, Alessandro Pancrazi^22^, Maria Lorubbio^22^, Massimo Vaghi^23^, Antonella D’Arminio Monforte^24^, Esther Merlini^24^, Federica Gaia Miraglia^24^, Raffaele Bruno^25,26^, Marco Vecchia^25^, Stefania Mantovani^25^, Serena Ludovisi^25,26^, Massimo Girardis^27^, Sophie Venturelli^27^, Marco Sita^27^, Andrea Cossarizza^28^, Andrea Antinori^29^, Alessandra Vergori^29^, Arianna Emiliozzi^29^, Stefano Rusconi^30,31^, Matteo Siano^31^, Arianna Gabrieli^31^, Agostino Riva^30,31^, Daniela Francisci^32,33^, Elisabetta Schiaroli^32^, Francesco Paciosi^32^, Andrea Tommasi^32^, Pier Giorgio Scotton^34^, Francesca Andretta^34^, Sandro Panese^35^, Renzo Scaggiante^36^, Francesca Gatti^36^, Saverio Giuseppe Parisi^37^, Melania degli Antoni^38^, Isabella Zanella^39,40^, Matteo Della Monica^41^, Carmelo Piscopo^41^, Mario Capasso^42,43,44^, Roberta Russo^42,43^, Immacolata Andolfo^42,43^, Achille Iolascon^42,43^, Giuseppe Fiorentino^45^, Massimo Carella^46^, Marco Castori^46^, Filippo Aucella^47^, Pamela Raggi^48^, Carmen Marciano^48^, Rita Perna^48^, Matteo Bassetti^49,50^, Antonio Di Biagio^49,50^, Maurizio Sanguinetti^51,52^, Luca Masucci^51,52^, Serafina Valente^53^, Marco Mandalà^54^, Alessia Giorli^54^, Lorenzo Salerni^54^, Patrizia Zucchi^55^, Pierpaolo Parravicini^55^, Elisabetta Menatti^56^, Stefano Baratti^57^, Tullio Trotta^58^, Ferdinando Giannattasio^58^, Gabriella Coiro^58^, Fabio Lena^59^, Domenico A. Coviello^60^, Cristina Mussini^61^, Giancarlo Bosio^62^, Enrico Martinelli^62^, Sandro Mancarella^63^, Luisa Tavecchia^63^, Mary Ann Belli^63^, Lia Crotti^64,65,66,67,68^, Gianfranco Parati^64,65^, Nicola Picchiotti^69,70^, Marco Gori^69,71^, Maurizio Sanarico^72^, Stefano Ceri^73^, Pietro Pinoli^73^, Francesco Raimondi^74^, Filippo Biscarini^75^, Alessandra Stella^75^, Marco Rizzi^76^, Franco Maggiolo^76^, Diego Ripamonti^76^, Claudia Suardi^77^, Tiziana Bachetti^78^, Maria Teresa La Rovere^79^, Simona Sarzi-Braga^80^, Maurizio Bussotti^81^, Katia Capitani^2,82^, Simona Dei^83^, Sabrina Ravaglia^84^, Rosangela Artuso^85^, Antonio Perrella^86^, Francesco Bianchi^2,86^, Giuseppe Merla^42,87^, Gabriella Maria Squeo^87^. 9. Dept of Specialized and Internal Medicine, Tropical and Infectious Diseases Unit, Azienda Ospedaliera Universitaria Senese, Siena, Italy; 10. Unit of Respiratory Diseases and Lung Transplantation, Department of Internal and Specialist Medicine, University of Siena; 11. Dept of Emergency and Urgency, Medicine, Surgery and Neurosciences, Unit of Intensive Care Medicine, Siena University Hospital, Italy; 12. Department of Medical, Surgical and Neurosciences and Radiological Sciences, Unit of Diagnostic Imaging, University of Siena; 13. Rheumatology Unit, Department of Medicine, Surgery and Neurosciences, University of Siena, Policlinico Le Scotte, Italy; 14. Department of Specialized and Internal Medicine, Infectious Diseases Unit, San Donato Hospital Arezzo, Italy; 15. Dept of Emergency, Anesthesia Unit, San Donato Hospital, Arezzo, Italy; 16. Department of Specialized and Internal Medicine, Pneumology Unit and UTIP, San Donato Hospital, Arezzo, Italy; 17. Department of Emergency, Anesthesia Unit, Misericordia Hospital, Grosseto, Italy; 18. Department of Specialized and Internal Medicine, Infectious Diseases Unit, Misericordia Hospital, Grosseto, Italy; 19. Laboratory Medicine Department, Misericordia Hospital, Grosseto, Italy; 20. Department of Preventive Medicine, Azienda USL Toscana Sud Est, Italy; 21. Territorial Scientific Technician Department, Azienda USL Toscana Sud Est, Italy; 22. Laboratory Medicine Department, San Donato Hospital, Arezzo, Italy; 23. Chirurgia Vascolare, Ospedale Maggiore di Crema, Italy; 24. Department of Health Sciences, Clinic of Infectious Diseases, ASST Santi Paolo e Carlo, University of Milan, Italy; 25. Division of Infectious Diseases and Immunology, Fondazione IRCCS Policlinico San Matteo, Pavia, Italy; 26. Department of Internal Medicine and Therapeutics, University of Pavia, Italy; 27. Department of Anesthesia and Intensive Care, University of Modena and Reggio Emilia, Modena, Italy; 28. Department of Medical and Surgical Sciences for Children and Adults, University of Modena and Reggio Emilia, Modena, Italy; 29. HIV/AIDS Department, National Institute for Infectious Diseases, IRCCS, Lazzaro Spallanzani, Rome, Italy; 30. III Infectious Diseases Unit, ASST-FBF-Sacco, Milan, Italy; 31. Department of Biomedical and Clinical Sciences Luigi Sacco, University of Milan, Milan, Italy; 32. Infectious Diseases Clinic, Department of Medicine, Azienda Ospedaliera di Perugia and University of Perugia, Santa Maria Hospital, Perugia, Italy; 33. Infectious Diseases Clinic, “Santa Maria” Hospital, University of Perugia, Perugia, Italy; 34. Department of Infectious Diseases, Treviso Hospital, Local Health Unit 2 Marca Trevigiana, Treviso, Italy; 35. Clinical Infectious Diseases, Mestre Hospital, Venezia, Italy; 36. Infectious Diseases Clinic, ULSS1, Belluno, Italy; 37. Department of Molecular Medicine, University of Padova, Italy; 38. Department of Infectious and Tropical Diseases, University of Brescia and ASST Spedali Civili Hospital, Brescia, Italy; 39. Department of Molecular and Translational Medicine, University of Brescia, Italy; 40. Clinical Chemistry Laboratory, Cytogenetics and Molecular Genetics Section, Diagnostic Department, ASST Spedali Civili di Brescia, Italy; 41. Medical Genetics and Laboratory of Medical Genetics Unit, A.O.R.N. “Antonio Cardarelli”, Naples, Italy; 42. Department of Molecular Medicine and Medical Biotechnology, University of Naples Federico II, Naples, Italy; 43. CEINGE Biotecnologie Avanzate, Naples, Italy; 44. IRCCS SDN, Naples, Italy; 45. Unit of Respiratory Physiopathology, AORN dei Colli, Monaldi Hospital, Naples, Italy; 46. Division of Medical Genetics, Fondazione IRCCS Casa Sollievo della Sofferenza Hospital, San Giovanni Rotondo, Italy; 47. Department of Medical Sciences, Fondazione IRCCS Casa Sollievo della Sofferenza Hospital, San Giovanni Rotondo, Italy; 48. Clinical Trial Office, Fondazione IRCCS Casa Sollievo della Sofferenza Hospital, San Giovanni Rotondo, Italy; 49. Department of Health Sciences, University of Genova, Genova, Italy; 50. Infectious Diseases Clinic, Policlinico San Martino Hospital, IRCCS for Cancer Research Genova, Italy; 51. Microbiology, Fondazione Policlinico Universitario Agostino Gemelli IRCCS, Catholic University of Medicine, Rome, Italy; 52. Department of Laboratory Sciences and Infectious Diseases, Fondazione Policlinico Universitario A. Gemelli IRCCS, Rome, Italy; 53. Department of Cardiovascular Diseases, University of Siena, Siena, Italy; 54. Otolaryngology Unit, University of Siena, Italy; 55. Department of Internal Medicine, ASST Valtellina e Alto Lario, Sondrio, Italy; 56. Study Coordinator Oncologia Medica e Ufficio Flussi, Sondrio, Italy; 57. Department of Infectious and Tropical Diseases, University of Padova, Padova, Italy; 58. First Aid Department, Luigi Curto Hospital, Polla, Salerno, Italy; 59. Local Health Unit-Pharmaceutical Department of Grosseto, Toscana Sud Est Local Health Unit, Grosseto, Italy; 60. U.O.C. Laboratorio di Genetica Umana, IRCCS Istituto G. Gaslini, Genova, Italy; 61. Infectious Diseases Clinics, University of Modena and Reggio Emilia, Modena, Italy; 62. Department of Respiratory Diseases, Azienda Ospedaliera di Cremona, Cremona, Italy; 63. U.O.C. Medicina, ASST Nord Milano, Ospedale Bassini, Cinisello Balsamo (MI), Italy; 64. Istituto Auxologico Italiano, IRCCS, Department of Cardiovascular, Neural and Metabolic Sciences, San Luca Hospital, Milan, Italy; 65. Department of Medicine and Surgery, University of Milano-Bicocca, Milan, Italy; 66. Istituto Auxologico Italiano, IRCCS, Center for Cardiac Arrhythmias of Genetic Origin, Milan, Italy; 67. Istituto Auxologico Italiano, IRCCS, Laboratory of Cardiovascular Genetics, Milan, Italy; 68. Member of the European Reference Network for Rare, Low Prevalence and Complex Diseases of the Heart-ERN GUARD-Heart; 69. University of Siena, DIISM-SAILAB, Siena, Italy; 70. Department of Mathematics, University of Pavia, Pavia, Italy; 71. University Cote d’Azur, Inria, CNRS, I3S, Maasai; 72. Independent Data Scientist, Milan, Italy; 73. Department of Electronics, Information and Bioengineering (DEIB), Politecnico di Milano, Milano, Italy; 74. Scuola Normale Superiore, Pisa, Italy; 75. CNR-Consiglio Nazionale delle Ricerche, Istituto di Biologia e Biotecnologia Agraria (IBBA), Milano, Italy; 76. Unit of Infectious Diseases, ASST Papa Giovanni XXIII Hospital, Bergamo, Italy; 77. Fondazione per la ricerca Ospedale di Bergamo, Bergamo, Italy; 78. Direzione Scientifica, Istituti Clinici Scientifici Maugeri IRCCS, Pavia, Italy; 79. Istituti Clinici Scientifici Maugeri IRCCS, Department of Cardiology, Institute of Montescano, Pavia, Italy; 80. Istituti Clinici Scientifici Maugeri, IRCCS, Department of Cardiac Rehabilitation, Institute of Tradate (VA), Italy; 81. Istituti Clinici Scientifici Maugeri IRCCS, Department of Cardiology, Institute of Milan, Milan, Italy; 82. Core Research Laboratory, ISPRO, Florence, Italy; 83. Health Management, Azienda USL Toscana Sudest, Tuscany, Italy; 84. IRCCS C. Mondino Foundation, Pavia, Italy; 85. Medical Genetics Unit, Meyer Children’s University Hospital, Florence, Italy; 86. Department of Medicine, Pneumology Unit, Misericordia Hospital, Grosseto, Italy; 87. Laboratory of Regulatory and Functional Genomics, Fondazione IRCCS Casa Sollievo della Sofferenza, San Giovanni Rotondo (Foggia), Italy.

## References

[1] Lopes-Pacheco M., Silva P.L., Cruz F.F., Battaglini D., Robba C., Pelosi P., Morales M.M., Caruso Neves C., Rocco P.R.M. (2021). Pathogenesis of Multiple Organ Injury in COVID-19 and Potential Therapeutic Strategies. Front. Physiol..

[2] WHO Coronavirus Disease 2019 (COVID-19) Situation Report. https://www.who.int/emergencies/diseases/novel-coronavirus-2019/situation-reports.

[3] Oran D.P., Topol E.J. (2020). Prevalence of Asymptomatic SARS-CoV-2 Infection. Ann. Intern. Med..

[4] Wiersinga W.J., Rhodes A., Cheng A.C., Peacock S.J., Prescott H.C. (2020). Pathophysiology, Transmission, Diagnosis, and Treatment of Coronavirus Disease 2019 (COVID-19). JAMA.

[5] Mahmudpour M., Roozbeh J., Keshavarz M., Farrokhi S., Nabipour I. (2020). COVID-19 cytokine storm: The anger of inflammation. Cytokine.

[6] Wang Y., Liu S., Liu H., Li W., Lin F., Jiang L., Li X., Xu P., Zhang L., Zhao L. (2020). SARS-CoV-2 infection of the liver directly contributes to hepatic impairment in patients with COVID-19. J. Hepatol..

[7] Wang F., Wang H., Fan J., Zhang Y., Wang H., Zhao Q. (2020). Pancreatic Injury Patterns in Patients With Coronavirus Disease 19 Pneumonia. Gastroenterology.

[8] Huang C., Wang Y., Li X., Ren L., Zhao J., Hu Y., Zhang L., Fan G., Xu J., Gu X. (2020). Clinical features of patients infected with 2019 novel coronavirus in Wuhan, China. Lancet (Lond. Engl.).

[9] Mao L., Jin H., Wang M., Hu Y., Chen S., He Q., Chang J., Hong C., Zhou Y., Wang D. (2020). Neurologic Manifestations of Hospitalized Patients With Coronavirus Disease 2019 in Wuhan, China. JAMA Neurol..

[10] Grasselli G., Greco M., Zanella A., Albano G., Antonelli M., Bellani G., Bonanomi E., Cabrini L., Carlesso E., Castelli G. (2020). Risk Factors Associated with Mortality among Patients with COVID-19 in Intensive Care Units in Lombardy, Italy. JAMA Intern. Med..

[11] Ellinghaus D., Degenhardt F., Bujanda L., Buti M., Albillos A., Invernizzi P., Fernández J., Prati D., Baselli G., Severe Covid-19 GWAS Group (2020). Genomewide Association Study of Severe Covid-19 with Respiratory Failure. N. Engl. J. Med..

[12] Vietzen H., Zoufaly A., Traugott M., Aberle J., Aberle S.W., Puchhammer-Stöckl E. (2021). Deletion of the NKG2C receptor encoding KLRC2 gene and HLA-E variants are risk factors for severe COVID-19. Genet. Med..

[13] Zhang Q., Liu Z., Moncada-Velez M., Chen J., Ogishi M., Bigio B., Yang R., Arias A.A., Zhou Q., Han J.E. (2020). Inborn errors of type I IFN immunity in patients with life-threatening COVID-19. Science.

[14] COVID-19 Host Genetics Initiative (2020). The COVID-19 Host Genetics Initiative, a global initiative to elucidate the role of host genetic factors in susceptibility and severity of the SARS-CoV-2 virus pandemic. Eur. J. Hum. Genet..

[15] Benetti E., Giliberti A., Emiliozzi A., Valentino F., Bergantini L., Fallerini C., Anedda F., Amitrano S., Conticini E., Tita R. (2020). Clinical and molecular characterization of COVID-19 hospitalized patients. PLoS ONE.

[16] Benetti E., Tita R., Spiga O., Ciolfi A., Birolo G., Bruselles A., Doddato G., Giliberti A., Marconi C., Musacchia F. (2020). ACE2 gene variants may underlie interindividual variability and susceptibility to COVID-19 in the Italian population. Eur. J. Hum. Genet..

[17] Pairo-Castineira E., Clohisey S., Klaric L., Bretherick A.D., Rawlik K., Pasko D., Walker S., Parkinson N., Fourman M.H., Russell C.D. (2020). Genetic mechanisms of critical illness in Covid-19. Nature.

[18] Daga S., Fallerini C., Baldassarri M., Fava F., Valentino F., Doddato G., Benetti E., Furini S., Giliberti A., Tita R. (2021). Employing a systematic approach to biobanking and analyzing clinical and genetic data for advancing COVID-19 research. Eur. J. Hum. Genet..

[19] Baldassarri M., Picchiotti N., Fava F., Fallerini C., Benetti E., Daga S., Valentino F., Doddato G., Furini S., Giliberti A. (2021). Shorter androgen receptor polyQ alleles protect against life-threatening COVID-19 disease in European males. EBioMedicine.

[20] Fallerini C., Daga S., Mantovani S., Benetti E., Picchiotti N., Francisci D., Paciosi F., Schiaroli E., Baldassarri M., Fava F. (2021). Association of toll-like receptor 7 variants with life-threatening COVID-19 disease in males: Findings from a nested case-control study. Elife.

[21] Elborn J.S. (2016). Cystic fibrosis. Lancet.

[22] Hoffmann M., Kleine-Weber H., Schroeder S., Krüger N., Herrler T., Erichsen S., Schiergens T.S., Herrler G., Wu N.-H., Nitsche A. (2020). SARS-CoV-2 Cell Entry Depends on ACE2 and TMPRSS2 and Is Blocked by a Clinically Proven Protease Inhibitor. Cell.

[23] Hamming I., Timens W., Bulthuis M.L.C., Lely A.T., Navis G.J., van Goor H. (2004). Tissue distribution of ACE2 protein, the functional receptor for SARS coronavirus. A first step in understanding SARS pathogenesis. J. Pathol..

[24] Roesch E.A., Nichols D.P., Chmiel J.F. (2018). Inflammation in cystic fibrosis: An update. Pediatr. Pulmonol..

[25] Miller A.C., Comellas A.P., Hornick D.B., Stoltz D.A., Cavanaugh J.E., Gerke A.K., Welsh M.J., Zabner J., Polgreen P.M. (2020). Cystic fibrosis carriers are at increased risk for a wide range of cystic fibrosis-related conditions. Proc. Natl. Acad. Sci. USA.

[26] Polgreen P.M., Brown G.D., Hornick D.B., Ahmad F., London B., Stoltz D.A., Comellas A.P. (2018). CFTR heterozygotes are at increased risk of respiratory infections: A population-based study. Open Forum Infect. Dis..

[27] (2020). WHO R&D Blueprint novel Coronavirus COVID-19 Therapeutic Trial Synopsis. https://www.who.int/publications/i/item/covid-19-therapeutic-trial-synopsis.

[28] Freeman G.H., Halton J.H. (1951). Note on an Exact Treatment of Contingency, Goodness of Fit and Other Problems of Significance. Biometrika.

[29] Groman J.D., Hefferon T.W., Casals T., Bassas L., Estivill X., Des Georges M., Guittard C., Koudova M., Fallin M.D., Nemeth K. (2004). Variation in a Repeat Sequence Determines Whether a Common Variant of the Cystic Fibrosis Transmembrane Conductance Regulator Gene Is Pathogenic or Benign. Am. J. Hum. Genet..

[30] Gabriel S.E., Brigman K.N., Koller B.H., Boucher R.C., Stutts M.J. (1994). Cystic fibrosis heterozygote resistance to cholera toxin in the cystic fibrosis mouse model. Science.

[31] Trapnell B.C., Chu C.S., Paakko P.K., Banks T.C., Yoshimura K., Ferrans V.J., Chernick M.S., Crystal R.G. (1991). Expression of the cystic fibrosis transmembrane conductance regulator gene in the respiratory tract of normal individuals and individuals with cystic fibrosis. Proc. Natl. Acad. Sci. USA..

[32] Cohn J.A., Friedman K.J., Noone P.G., Knowles M.R., Silverman L.M., Jowell P.S. (1998). Relation between Mutations of the Cystic Fibrosis Gene and Idiopathic Pancreatitis. N. Engl. J. Med..

[33] Peckham H., de Gruijter N.M., Raine C., Radziszewska A., Ciurtin C., Wedderburn L.R., Rosser E.C., Webb K., Deakin C.T. (2020). Male sex identified by global COVID-19 meta-analysis as a risk factor for death and ITU admission. Nat. Commun..

[34] Jin J.M., Bai P., He W., Wu F., Liu X.F., Han D.M., Liu S., Yang J.K. (2020). Gender Differences in Patients With COVID-19: Focus on Severity and Mortality. Front. Public Health.

[35] Verma S., Saksena S., Sadri-Ardekani H. (2020). ACE2 receptor expression in testes: Implications in coronavirus disease 2019 pathogenesis. Biol. Reprod..

[36] Tay M.Z., Poh C.M., Rénia L., MacAry P.A., Ng L.F.P. (2020). The trinity of COVID-19: Immunity, inflammation and intervention. Nat. Rev. Immunol..

[37] Kuba K., Imai Y., Rao S., Gao H., Guo F., Guan B., Huan Y., Yang P., Zhang Y., Deng W. (2005). A crucial role of angiotensin converting enzyme 2 (ACE2) in SARS coronavirus-induced lung injury. Nat. Med..

[38] McNabb-Baltar J., Jin D.X., Grover A.S., Redd W.D., Zhou J.C., Hathorn K.E., McCarty T.R., Bazarbashi A.N., Shen L., Chan W.W. (2020). Lipase Elevation in Patients With COVID-19. Am. J. Gastroenterol..

[39] Sarantis P., Koustas E., Papavassiliou A.G., Karamouzis M.V. (2020). Are cystic fibrosis mutation carriers a potentially highly vulnerable group to COVID-19?. J. Cell. Mol. Med..

[40] Shah V.S., Ernst S., Tang X.X., Karp P.H., Parker C.P., Ostedgaard L.S., Welsh M.J. (2016). Relationships among CFTR expression, HCO3- secretion, and host defense may inform gene- and cell-based cystic fibrosis therapies. Proc. Natl. Acad. Sci. USA.

[41] Bezzerri V., Lucca F., Volpi S., Cipolli M. (2020). Does cystic fibrosis constitute an advantage in COVID-19 infection?. Ital. J. Pediatr..

[42] Colombo C., Burgel P.-R., Gartner S., van Koningsbruggen-Rietschel S., Naehrlich L., Sermet-Gaudelus I., Southern K.W. (2020). Impact of COVID-19 on people with cystic fibrosis. Lancet. Respir. Med..

[43] Cosgriff R., Ahern S., Bell S.C., Brownlee K., Burgel P.-R., Byrnes C., Corvol H., Cheng S.Y., Elbert A., Faro A. (2020). A multinational report to characterise SARS-CoV-2 infection in people with cystic fibrosis. J. Cyst. Fibros..

[44] Chen H.H., Shaw D.M., Petty L.E., Graff M., Bohlender R.J., Polikowsky H.G., Zhong X., Kim D., Buchanan V.L., Preuss M.H. (2020). Host genetic effects in pneumonia. Am. J. Hum. Genet..

[45] Chen R., Liang W., Jiang M., Guan W., Zhan C., Wang T., Tang C., Sang L., Liu J., Ni Z. (2020). Risk Factors of Fatal Outcome in Hospitalized Subjects With Coronavirus Disease 2019 From a Nationwide Analysis in China. Chest.

[46] Price-Haywood E.G., Burton J., Fort D., Seoane L. (2020). Hospitalization and Mortality among Black Patients and White Patients with Covid-19. N. Engl. J. Med..

[47] Castellani C., Massie J., Sontag M., Southern K.W. (2016). Newborn screening for cystic fibrosis. Lancet Respir. Med..

