# Supplementary material for: Severe COVID-19 in Hospitalized Carriers of Single CFTR Pathogenic Variants
Source: J Pers Med. 2021 Jun 15;11(6):558. doi: 10.3390/jpm11060558 (PMC8232773; doi:10.3390/jpm11060558)
Supplement: Supplementary file 1 [file jpm-11-00558-s001.zip › Supp Table 1_ok.pdf]

Table S1. Prevalence of comorbidities by COVID-19 outcome scale and sex

Chronic Conditions, No. (%)	Whole Cohort (n=874)	Female (n=377)	Male (n=496)
**Hypertension**			
Cat. 1 (Death; n=55)	14 (25.45) **	7 (50)	7 (50)
Cat. 2 (Invasive mechanical ventilation; n=70)	31 (44.29) **	9 (29.03)	22 (70.97)
Cat. 3 (CPAP/BiPAP; n=146)	52 (35.62) **	13 (25)	39 (75)
Cat. 4 (Hospitalized, receiving supplemental low flow oxygen; n=276)	112 (40.58) **	49 (43.75)	63 (56.25)
Cat. 5 (Hospitalized, not receiving supplemental oxygen; n=122)	23 (18.85) *	10 (43.48)	13 (56.52)
Cat. 6 (Not hospitalized; n=205)	11 (5.36)	5 (45.45)	6 (54.55)
**Diabetes**			
Cat. 1 (Death; n=55)	8 (14.55) **	2 (25)	6 (75)
Cat. 2 (Invasive mechanical ventilation; n=70)	11 (15.71) **	4 (36.36)	7 (63.64)
Cat. 3 (CPAP/BiPAP; n=146)	24 (16.44) **	8 (33.33)	16 (66.67)
Cat. 4 (Hospitalized, receiving supplemental low flow oxygen; n=276)	47 (17.03) **	20 (42.55)	27 (57.45)
Cat. 5 (Hospitalized, not receiving supplemental oxygen; n=122)	10 (8.2) *	6 (60)	4 (40)
Cat. 6 (Not hospitalized; n=205)	5 (2.44)	4 (20)	1 (20)
**Asthma and COPD**			
Cat. 1 (Death; n=55)	5 (9.09) §	3 (33.33)	2 (66.67)
Cat. 2 (Invasive mechanical ventilation; n=70)	13 (18.57) **	3 (23.08)	10 (76.92)
Cat. 3 (CPAP/BiPAP; n=146)	17 (11.64) **	7 (41.18)	10 (58.82)
Cat. 4 (Hospitalized, receiving supplemental low flow oxygen; n=276)	30 (10.87) **	16 (53.33)	14 (46.67)
Cat. 5 (Hospitalized, not receiving supplemental oxygen; n=122)	6 (4.92) §	4 (66.67)	2 (33.33)
Cat. 6 (Not hospitalized; n=205)	1 (0.49)	0	1 (100)
**CHF and CAD**			
Cat. 1 (Death; n=55)	8 (14.55) **	3 (37.50)	5 (62.5)
Cat. 2 (Invasive mechanical ventilation; n=70)	4 (5.71) §	1 (25)	3 (75)
Cat. 3 (CPAP/BiPAP; n=146)	12 (8.22) *	4 (33.33)	8 (66.67)
Cat. 4 (Hospitalized, receiving supplemental low flow oxygen; n=276)	33 (11.96) **	9 (27.27)	24 (72.73)
Cat. 5 (Hospitalized, not receiving supplemental oxygen; n=122)	9 (7.38) **	3 (33.33)	6 (66.67)
Cat. 6 (Not hospitalized; n=205)	2 (0.98)	1 (50)	1 (50)

Chronic Conditions, No. (%)	Whole Cohort (n=874)	Female (n=377)	Male (n=496)
**Malignancy**			
Cat. 1 (Death; n=55)	4 (7.27) *	2 (50)	2 (50)
Cat. 2 (Invasive mechanical ventilation; n=70)	5 (7.14)	1 (20)	4 (80)
Cat. 3 (CPAP/BiPAP; n=146)	15 (10.27) *	9 (60) °°	6 (40)
Cat. 4 (Hospitalized, receiving supplemental low flow oxygen; n=276)	22 (7.97) §	12 (54.55)	10 (45.45)
Cat. 5 (Hospitalized, not receiving supplemental oxygen; n=122)	13 (10.66) §	8 (61.54)	5 (38.46)
Cat. 6 (Not hospitalized; n=205)	6 (2.93)	3 (50)	3 (50)
**Hypothyroidism**			
Cat. 1 (Death; n=55)	3 (5.45)	3 (100)	0
Cat. 2 (Invasive mechanical ventilation; n=70)	5 (7.14)	3 (60)	2 (40)
Cat. 3 (CPAP/BiPAP; n=146)	7 (4.79)	3 (42.86)	4 (57.14)
Cat. 4 (Hospitalized, receiving supplemental low flow oxygen; n=276)	13 (4.71)	9 (69.23) °	4 (30.77)
Cat. 5 (Hospitalized, not receiving supplemental oxygen; n=122)	8 (6.56)	6 (75)	2 (25)
Cat. 6 (Not hospitalized; n=205)	6 (2.93)	5 (83.33)	1 (16.67)
**Obesity**			
Cat. 1 (Death; n=55)	2 (3.64)	1 (50)	1 (50)
Cat. 2 (Invasive mechanical ventilation; n=70)	10 (14.29) **	3 (30)	7 (70)
Cat. 3 (CPAP/BiPAP; n=146)	5 (3.42)	1 (20)	4 (80)
Cat. 4 (Hospitalized, receiving supplemental low flow oxygen; n=276)	8 (2.9)	6 (75)	2 (25)
Cat. 5 (Hospitalized, not receiving supplemental oxygen; n=122)	3 (2.46)	1 (33.33)	2 (66.67)
Cat. 6 (Not hospitalized; n=205)	1 (0.49)	1 (100)	0

CPAP: Continuous Positive Airway Pressure; BiPAP: Bilevel Positive Airway Pressure; CHF: Congestive Heart Failure; CAD: Coronary Artery Disease; COPD: Chronic Obstructive Pulmonary Disease. Percentage in column 2 indicates the prevalence in the whole cohort while in column 3 and 4 it refers to the prevalence of males and females in the group indicated in the corresponding line (i.e. COVID-19 category of chronic condition). Fisher exact test was performed. **p<0.001; *p<0.01; §p<0.05 vs Cat. 6 - °p<0.05; °°p<0.01 vs other sex.
