# Supplementary material for: Severe COVID-19 in Hospitalized Carriers of Single CFTR Pathogenic Variants
Source: J Pers Med. 2021 Jun 15;11(6):558. doi: 10.3390/jpm11060558 (PMC8232773; doi:10.3390/jpm11060558)
Supplement: Supplementary file 1 [file jpm-11-00558-s001.zip › Supp Table 2_ok.pdf]

Table S2. CF causing variants in COVID-19 patients

**Patient**	**Gender**	**Age (years)**	**COVID-19 Outcome scale Category**	**Chronic Conditions**	**CF-causing *CFTR* variants**	**Other *CFTR* rare variants**	**Val470Met common variant**
**Carriers of 1 CF-causing variant**							
**1** ^	M	72	1	--	Gly1069Arg	Asn417Lys	homo
**2**	F	64	1	Obesity	Asn1303Lys	--	homo
**3**	F	84	1	Hypotiroidism, CHD, Neurological disease	Phe1052Val	--	hetero
**4**	M	86	1	AF, Parkinson Disease	Phe508del	--	hetero
**5**	M	32	2	--	Phe508del	Arg347Cys	homo
**6**	M	37	2	--	Leu967Ser		hetero
**7**	M	69	2	--	Phe508del		homo
**8**	M	30	2	--	Phe508del	Ala238Val	homo
**9**	F	75	2	Hypertension	Phe1052Val	--	hetero
**10**	M	63	2	HIV	c.1585-1G>A	--	hetero
**11**	M	36	3	Asthma	Phe508del	--	hetero
**12**	M	89	3	Asthma, CKD	Phe508del	--	hetero
**13**	F	78	3	CHF, Malignancy, DM, COPD	Ser1455*	--	--
**14**	M	53	3	CHD	Gly1069Arg	--	--
**15**	F	77	3	--	Gly542*	Val562Ile	homo
**16**	F	76	3	CHD, Dyslipidemia	Phe508del	--	homo
**17**	F	51	3	Malignancy	Phe508del	--	hetero
**18**	M	46	3	--	Trp1282*	--	hetero
**19**	M	52	4	--	Ser945Leu	--	hetero
**20**	F	31	4	--	Leu967Ser	--	--
**21**	M	66	4	Hypertension, Dyslipidemia	Arg347Pro	--	--
**22**	M	63	4	Hypertension, Dyslipidemia	c.1585-1G>A	--	hetero
**23**	M	47	4	--	Arg1066His	--	homo
**24**	F	54	4	--	Gln39*	--	hetero
**25**	M	88	4	CHF, AF, aortic valve replacement, hypertension, CKD, hyperuricemia	Thr338Ile	--	hetero
**26**	M	67	5		Arg347Pro	--	--
**27**	M	60	5	CHF	Gly1069Arg	--	--
**28**	F	59	5	Leukoencephalopathy	Gly1349Asp	--	hetero
**29** ^	F	46	5	--	Leu967Ser	--	--

Patient	Gender	Age (years)	COVID-19 Outcome scale Category	Chronic Conditions	CF-causing *CFTR* variants	Other *CFTR* rare variants	Val470Met common variant
**30**	M	45	5	--	Phe508del	--	hetero
**31**	M	47	6	-	Leu967Ser	--	--
**32**	F	60	6	Asthma	Phe508del	--	hetero
**33**	M	28	6		Phe508del	Gly576Ala; Arg668Cys	homo
**34**	F	51	6	--	Arg1162*	--	hetero
**35**	M	31	6	Brugada syndrome	Phe508del	--	homo
**36**	M	43	6	--	Phe508del	--	hetero
**37**	M	60	6	--	Gly1069Arg	--	--
**38**	M	21	6	DM, mild ID	Gly542*	Asn417Lys	homo
**39**	M	49	6	--	Gly1069Arg	--	--
**40**	F	48	6	--	Asn1303Thrfs*25	--	hetero
**Carrier of 2 CF-causing variants**							
**1**	M	52	4	CHD	Phe508del; Phe1052Val	--	homo

COVID-19 Outcome Scale categories: 1, death; 2, hospitalized receiving invasive mechanical ventilation; 3, hospitalized, receiving Continuous positive airway pressure (CPAP) or Bilevel Positive Airway Pressure (BiPAP) ventilation; 4, hospitalized, receiving supplemental oxygen without positive pressure or high flow; 5, hospitalized, not receiving supplemental oxygen; 6, not hospitalized. CHD: Coronary heart disease; AF: Atrial Fibrillation; CKD: Chronic Kidney Disease; CHF: Congestive Heart Failure; DM: Diabetes Mellitus; COPD: Chronic Obstructive Pulmonary Disease; ID: Intellectual Disability. ^: patient carrying 11GT-5T/10TG-7T polymorphism.
