# Supplementary material for: Severe COVID-19 in Hospitalized Carriers of Single CFTR Pathogenic Variants
Source: J Pers Med. 2021 Jun 15;11(6):558. doi: 10.3390/jpm11060558 (PMC8232773; doi:10.3390/jpm11060558)
Supplement: Supplementary file 1 [file jpm-11-00558-s001.zip › Supp Table 3_ok.pdf]

Table S3. Carriers of *CFTR* pathogenic variants by COVID-19 outcome scale and sex

Carriers of 1 CF-causing variant		
	**Female (n=14, 35%)**	**Male (n=26, 65%)**
**COVID Outcome Scale, No. (%)**		
Cat. 1 (Death)	2 (50%)	2 (50%)
Cat. 2 (Invasive mechanical ventilation)	1 (16.67%)	5 (83.33%)*
Cat. 3 (CPAP/BiPAP)	4 (50%)	4 (50%)
Cat. 4 (Hospitalized with low flow oxygen)	2 (28.57%)	5 (71.43%)*
Cat. 5 (Hospitalized without oxygen)	2 (40%)	3 (60%)*
Cat. 6 (Not hospitalized)	3 (30%)	7 (70%)*
**Carrier of 2 CF-causing variants**		
	**Female (n=0)**	**Male (n=1, 100%)**
Cat. 4 (Hospitalized with low flow oxygen)	0	1 (100%)

*$p<0.05$ by Fisher exact test

CPAP: Continuous Positive Airway Pressure; BiPAP: Bilevel Positive Airway Pressure. Percentage in column 2 and 3 refers to the prevalence of males and females in the corresponding COVID-19 category.
