# Supplementary material for: Severe COVID-19 in Hospitalized Carriers of Single CFTR Pathogenic Variants
Source: J Pers Med. 2021 Jun 15;11(6):558. doi: 10.3390/jpm11060558 (PMC8232773; doi:10.3390/jpm11060558)
Supplement: Supplementary file 1 [file jpm-11-00558-s001.zip › Supp Table 4_ok.pdf]

Table S4. Presence of comorbidities by carrier status

	**Not Carriers (n=833)**	**Carriers (n=40)**
**Chronic Conditions, No. (%)**		
Hypertension	239 (28.69) *	4 (10)
Diabetes	103 (12.36)	2 (5)
Asthma and COPD	69 (8.28)	3 (7.5)
CHF and CAD	61 (7.32)	6 (15)
Malignancy	63 (7.56)	2 (5)
Hypothyroidism	41 (4.92)	1 (2.5)
Obesity	28 (3.36)	1 (2.5

*p<0.05 vs carriers by Fisher exact test. CHF: Congestive Heart Failure; CAD: Coronary Artery Disease; COPD: Chronic Obstructive Pulmonary Disease.
