# Supplementary material for: Severe COVID-19 in Hospitalized Carriers of Single CFTR Pathogenic Variants
Source: J Pers Med. 2021 Jun 15;11(6):558. doi: 10.3390/jpm11060558 (PMC8232773; doi:10.3390/jpm11060558)
Supplement: Supplementary file 1 [file jpm-11-00558-s001.zip › Supp Table 5_ok.pdf]

Table S5. Univariable Cox analysis and risk factors related to fatal outcome

Variable	Level	HR	95% CI	Wald test p
**14-day fatal outcome**				
Sex	female vs male	1.46	0.74-2.9	0.2742
Age	≥75 years	4.53	2.27-9.04	<0.0001
Age	≥65 years	5.78	2.23-14.98	0.0003
Blood type A	yes vs no	1.21	0.55-2.65	0.6374
**Genetic profile**				
Carrier of one CF-causing variant	yes vs no	2.86	1.01-8.15	0.0480
**Comorbidities**				
Diabetes	yes vs no	0.85	0.30-2.42	0.7614
Malignancy	yes vs no	0.64	0.15-2.67	0.5408
CHF and CAD	yes vs no	1.31	0.46-3.72	0.6163
Hypertension	yes vs no	0.60	0.27-1.34	0.2135
Asthma and COPD	yes vs no	1.14	0.40-3.25	0.8012
**Serum Biochemistry**				
C-reactive protein (mg/dl)	≥20	1.50	0.70-3.21	0.2951
Aspartate Aminotransferase (U/L)	≥40	1.52	0.65-3.55	0.3367
Alanine Aminotransferase (U/L)	≥40	0.43	0.19-0.97	0.0414
Lactic Acid Dehydrogenase (U/L)	≥400	3.64	1.3-10.22	0.0141
**28-day fatal outcome**				
Sex	female vs male	1.18	0.65-2.14	0.5864
Age	≥75 years	4.00	2.21-7.24	<0.0001
Age	≥65 years	5.47	2.44-12.27	<0.0001
Blood type A	yes vs no	0.97	0.49-1.90	0.9233
**Genetic profile**				
Carrier of one CF-causing variant	yes vs no	1.97	0.7-5.5	0.1978
**Comorbidities**				
Diabetes	yes vs no	0.79	0.31-1.99	0.6124
Malignancy	yes vs no	0.71	0.22-2.29	0.5679
CHF and CAD	yes vs no	1.25	0.49-3.17	0.6374
Hypertension	yes vs no	0.61	0.31-1.21	0.1577
Asthma and COPD	yes vs no	0.88	0.31-2.45	0.8012
**Serum Biochemistry**				
C-reactive protein (mg/dl)	≥20	1.35	0.71-2.60	0.3608
Aspartate Aminotransferase (U/L)	≥40	1.96	0.91-4.23	0.0846
Alanine Aminotransferase (U/L)	≥40	0.59	0.30-1.16	0.1273
Lactic Acid Dehydrogenase (U/L)	≥400	3.57	1.50-8.51	0.0040

60-day fatal outcome				
Variable	**Level**	**HR**	**95% CI**	**Wald test p**
Sex	female vs male	1.12	0.64-1.96	0.6984
Age	≥75 years	3.33	1.91-5.8	<0.0001
Age	≥65 years	4.71	2.29-9.68	<0.0001
Blood type A	yes vs no	1.06	0.57-1.97	0.8510
**Genetic profile**				
Carrier of one CF-causing variant	yes vs no	1.70	0.61-4.73	0.3087
**Comorbidities**				
Diabetes	yes vs no	0.97	0.44-2.17	0.9476
Malignancy	yes vs no	0.64	0.20-2.04	0.4466
CHF and CAD	yes vs no	1.58	0.71-3.5	0.2644
Hypertension	yes vs no	0.55	0.29-1.06	0.0741
Asthma and COPD	yes vs no	0.95	0.38-2.41	0.9212
**Serum Biochemistry**				
C-reactive protein (mg/dl)	≥20	1.33	0.72-2.45	0.3660
Aspartate Aminotransferase (U/L)	≥40	2.25	1.06-4.76	0.0342
Alanine Aminotransferase (U/L)	≥40	0.71	0.37-1.37	0.3066
Lactic Acid Dehydrogenase (U/L)	≥400	3.56	1.59-7.98	0.0020
